# Quantification of Choroidal Vascular Hyperpermeability on Ultra-Widefield Indocyanine Green Angiography in Macular Neovascularization

**DOI:** 10.3390/diagnostics14070754

**Published:** 2024-04-02

**Authors:** Ho Ra, Younhea Jung, Seung Hoon Lee, Seo-woo Park, Jay Chhablani, Jiwon Baek

**Affiliations:** 1 Department of Ophthalmology, Bucheon St. Mary’s Hospital, College of Medicine, The Catholic University of Korea, Bucheon 14647, Republic of Korea; raho@catholic.ac.kr (H.R.); 1124mynamelsh@gmail.com (S.H.L.); psw961001@gmail.com (S.-w.P.); 2 Department of Ophthalmology, College of Medicine, The Catholic University of Korea, Seoul 06591, Republic of Korea; write2une@catholic.ac.kr; 3 Department of Ophthalmology, Yeoui-do St. Mary’s Hospital, College of Medicine, The Catholic University of Korea, Seoul 07345, Republic of Korea; 4 Medical Retina and Vitreoretinal Surgery, University of Pittsburgh School of Medicine, Pittsburg, PA 15261, USA; jay.chhablani@gmail.com

**Keywords:** choroidal vascular hyperpermeability, quantification, ultra-widefield indocyanine green angiography, ICGA

## Abstract

To obtain a quantitative parameter for the measurement of choroidal vascular hyperpermeability (CVH) on ultra-widefield indocyanine green angiography (UWICGA) using an objective analysis method in macular choroidal neovascularization (CNV). A total of 113 UWICGA images from 113 subjects were obtained, including with 25 neovascular age-related macular degeneration (nAMD), 37 with polypoidal choroidal vasculopathy (PCV) (19 with thin-choroid and 18 with thick-choroid), 33 with pachychoroid neovasculopathy (PNV), and 18 age-matched controls. CVH was quantified on a gray image by the subtraction of 2 synchronized UWICGA images of early and late phases. The measured CVH parameter was compared with human graders and among CNV subtypes and correlated with choroidal vascular density (CVD) and subfoveal choroidal thickness (SFCT). The mean CVH values were 28.58 ± 4.97, 33.36 ± 8.40, 33.61 ± 11.50, 42.19 ± 13.25, and 43.59 ± 7.86 in controls and patients with nAMD, thin-choroid PCV, thick-choroid PCV, and PNV, respectively (*p* < 0.001). CVH was higher in thick-choroid PCV and PNV compared to the other groups (all *p* ≤ 0.006). The measured CVH value positively correlated with those reported by human graders (*p* < 0.001), CVD, and SFCT (*p* = 0.001 and *p* < 0.001, respectively). CVH can be measured objectively using quantitative UWICGA analysis. The CVH parameter differs among macular CNV subtypes and correlates with CVD and SFCT.

## 1. Introduction

Choroidal vascular hyperpermeability (CVH) is a distinctive choroidal finding in some patients with macular disease. Central serous chorioretinopathy (CSC) is a representative of them. The first published description of CVH was in a patient with CSC [1]. In 1994, Piccolino et al. and Guyer et al. described localized hyperpermeability of the choriocapillaris on indocyanine green angiography (ICG) in CSC and presumed its association with the disease pathophysiology [1,2]. In addition to CSC, researchers found that CVH is also frequently present in patients with polypoidal choroidal vasculopathy (PCV) [3].

With the advances that have occurred in optical coherence tomography (OCT) imaging systems, more detailed observation of the choroid in vivo has been made possible. Differences in choroidal morphology on B-scan sechtions among macular diseases with CNV have been discovered. Some subtypes share similar choroidal features with CSC, including a pathologically thickened choroid with prominent Haller vessel dilatation accompanied by attenuated choriocapillaris and a Sattler layer [4,5]. These subtypes include PCV and pachychoroid neovasculopathy (PNV).

Thousands of studies have been carried out and published to quantitatively measure the choroidal thickness or choroidal vascular density (CVD) in this regard. On the contrary, although CVH is also a constant finding associated with disease pathophysiology in CSC and other macular diseases, an objective and quantitative method of CVH measurement has not been suggested yet.

The purpose of this study was therefore to obtain a quantitative parameter for the measurement of CVH on ultra-widefield ICGA (UWICGA) using an objective analysis method in macular CNV and to compare the measured CVH parameter with human graders to confirm its reliability. Also, a comparison of CVH values among macular CNV subtypes and correlations with other choroidal parameters, including CVD, were completed. 

## 2. Materials and Methods

This retrospective case–control study was conducted by a review of the medical records. Consecutive patients with typical neovascular age-related macular degeneration (nAMD), PCV, and PNV who visited Bucheon St. Mary’s Hospital (College of Medicine, the Catholic University of Korea, Gyeonggi-do, Republic of Korea) between April 2018 and July 2022 were subjected. Patients without retinal disease who underwent UWICGA imaging were included as controls. The study was approved by the institutional review board of the Hospital (HC21RASI0007) and waived the need for written informed consent due to the study’s retrospective design. The study was conducted in accordance with the tenets of the Declaration of Helsinki.

### 2.1. Patients

The diagnosis of nAMD was made based upon a combination of OCT, fluorescein angiography, and ICGA data suggesting evidence of CNV with leakage associated with pigment epithelial detachment, serous retinal detachment, subretinal exudation, and hemorrhage in the macular region. nAMD with type 2 CNV was included for the study. When the CNV was type 1 and associated with dilated Haller vessels below as detected by ICGA and OCT in patients without any features of typical AMD, it was defined as PNV. PCV was diagnosed by the presence of characteristic polypoidal vascular abnormalities with a branching vascular network as detected on OCT and ICGA. PCV cases were divided into thick- and thin-choroid PCV cases with a subfoveal choroidal thickness (SFCT) cutoff value of 300 μm [6]. In bilateral cases, one eye was randomly selected for enrollment at baseline.

The exclusion criteria were as follows: (1) a history of previous treatment for retinal disease that might affect the choroid (e.g., laser photocoagulation, photodynamic therapy, intraocular and/or periocular injections); (2) other concomitant eye conditions, including choroidal neovascularization other than the above criteria, high myopia (>−6.00 D or axial length > 26 mm), epiretinal membrane, macular hole, uveitis, diabetic retinopathy, or glaucoma; and (3) image quality degrading severe media opacity.

Data including age, sex, and Snellen best-corrected visual acuity (BCVA) were obtained from a chart review. Baseline optical coherence tomography (Cirrus-HD 4000 or 6000; Carl Zeiss Meditec, Jena, Germany), mydriatic ultra-widefield fluorescein angiography (UWFA) (Optos California P200DTx icg; Optos, Dunfermline, UK), and UWICGA (Optos California P200DTx icg, Dunfermline, UK) imaging were performed.

### 2.2. CVH Measurement

**Figure 1:**
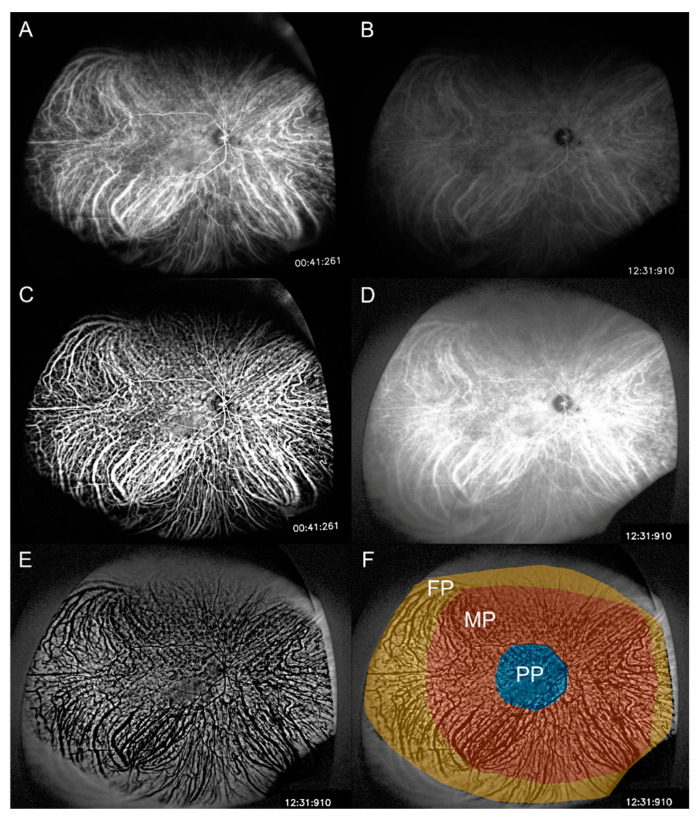
Measurement of choroidal vascular hyperpermeability (CVH) on ultra-widefield indocyanine green angiography (UWICGA). UWICGA images in the early phase (30–60 s after the dye injection) (**A**) and late phase (10–13 min after dye injection) (**B**) were saved as 1027- × 800-pixel 8-unit grayscale bitmap images (.bmp). Early UWICGA images were preprocessed using a homomorphic filter and contrast adjustment to obtain choroidal large vessels (**C**). Late UWICGA images were preprocessed to equalize the contrast level among subjects (**D**). Subtraction of (**C**) from (**B**) was performed to obtain the CVH value (**E**). CVH was measured as the total pixel intensity (0–255) divided by the total pixel area of the region of interest. Measurements were collected for the total gradable area, PP area, MP, and FP (**F**).

Two UWICGA images were saved as 8-unit grayscale bitmap images (.bmp) with 1027 × 800 pixels: the best one was taken 30–60 s after the dye injection (early UWICGA) and the other was taken 10–13 min after the dye injection (late UWICGA) (Figure 1A,B). Early UWICGA images were preprocessed using a homomorphic filter plugin in the FIJI software (an expanded version of ImageJ version 1.51a available at fiji.sc free of charge) and contrast adjustment using the “stretchlim” function in MATLAB 2022a (MathWorks, Inc., Natick, MA, USA) to best obtain choroidal large vessels (Figure 1C). Late UWICGA images were preprocessed using contrast adjustment to remove differences in the contrast level among subjects (Figure 1D). Then, image subtraction from the late UWICGA to early UWICGA period was done to obtain late leakage data, including CVH values (Figure 1E). The border of the gradable area was defined manually by a grading retina specialist. CVH was measured as a total pixel intensity (0–255 of the 8-unit grayscale image) divided by the total pixel area of the region of interest. The measurement was performed for the total gradable area, posterior polar (PP) area, mid-periphery (MP), and far-periphery (FP). The PP area was defined as the retina just beyond the disc and major arcades, the MP was defined as the area inside the line connecting visible vortex ampullae, and FP was defined as the rest of the gradable area outside the MP (Figure 1F) [7]. 

**Figure 2:**
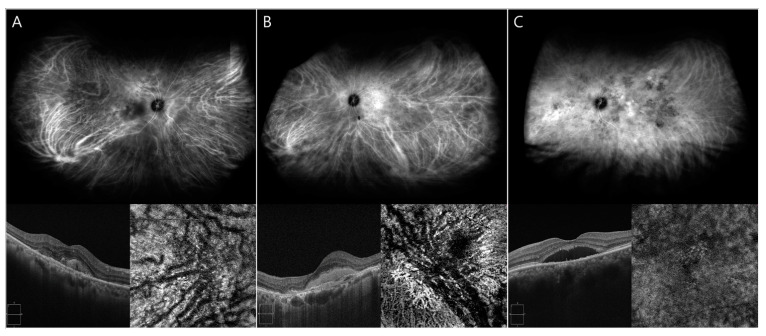
Representative cases of choroidal vascular hyperpermeability (CVH). Standard ultra-widefield indocyanine green angiographic (ICGA) images for the grading of choroidal vascular hyperpermeability (top row) and optical coherence tomography (OCT) and OCT angiography (bottom row) images. The CVH on the late phase ultra-widefield ICGA was graded as none, mild (**A**), moderate (**B**), or severe (**C**) based on the standard image by two retinal specialists.

CVH was graded subjectively on late-phase UWICGA images by two retina specialists as none (0), mild (1), moderate (2), or severe (3) based on standard UWICGA images defined by the agreement of other two retina specialists (Figure 2). The mean CVH value of both graders’ scores was used. 

### 2.3. Other Choroidal Parameter Measurements

On OCT, using horizontal line scans intersecting the center of the fovea, subfoveal choroidal thickness (SFCT) was measured as the distance between the Bruch membrane and the choroid–scleral border at the fovea [8].

CVD was measured on UWICGA images based on a previously described method with some modification [9]. Briefly, UWICGA images were binarized using the Midgrey method (radius, 30), and retinal vessels were subtracted with a mask obtained using early-phase (20–40 s) UWFA after alignment with UWICGA. Then, the CVD was calculated by dividing the number of pixels in the vascular area (white pixels) by that of the total gradable area. CVDs were also measured for the total area, PP area, MP, and FP, which were similar to the CVH measurements (Figure 1F).

#### Statistical Analysis

Statistical analysis was performed using SPSS version 26.0.1 for Windows (IBM Corp., Armonk, NY, USA). An analysis of variance (ANOVA) was performed to determine the differences among groups, and a post-hoc analysis was done using the least significant difference (LSD). A chi-squared test was used to compare categorical variables between groups. Repeated-measures ANOVA was used to compare 3 different areas. For the analysis of correlations between parameters, Pearson’s correlation coefficient was incorporated. The Kruskal–Wallis test was used to compare the CVH and CVD values of each area. Continuous variables are described as mean ± standard deviation values. BCVAs were converted to logarithm of minimal angle of resolution (logMAR) values. *p* < 0.05 was considered to be statistically significant.

## 3. Results

### 3.1. Demographics of the Study Subjects

A total of 113 eyes of 113 subjects—including 25 with nAMD, 19 with thin-choroid PCV, 18 with thick-choroid PCV, 33 with PNV, and 18 controls—were included in this study. All subjects were Koreans. The mean age of study participants was 68.80 ± 10.72 years, 62 (55%) were male, and 65 had right eyes enrolled (58%). The baseline logMAR BCVA value was 0.38 ± 0.48.

Age and sex distribution differed between groups (*p* = 0.038 and *p* = 0.040, respectively). Patients with nAMD or thin-choroid PCV were older than those with PNV (*p* = 0.003 and *p* = 0.032, respectively), without other significant differences in age found among groups (all *p* ≥ 0.059). The proportion of female subjects was greater in the nAMD group compared to the other groups (z-score = 3.1 in nAMD vs. −1.3, −0.6, and −1.2 in thin-choroid PCV, thick-choroid PCV, and PNV, respectively). Comparisons of demographics among and between groups are summarized in Table 1.

### 3.2. CVH Measured on UWICGA Images by Group

**Figure 3:**
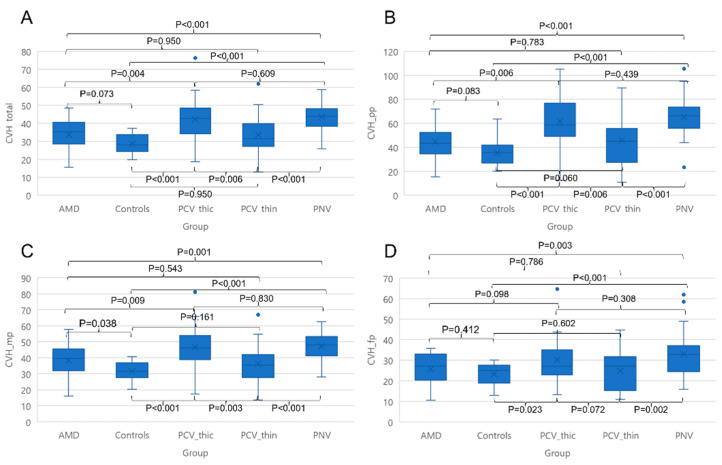
Choroidal vascular hyperpermeability (CVH) measured on ultra-widefield indocyanine green angiography (UWICGA) images by group. CVH on UWICGA images of the total gradable area (**A**), posterior pole (PP) area (**B**), mid-periphery (MP) (**C**), and far-periphery (FP) (**D**). UWICGA images of the total gradable area, PP area, and MP reveal higher CVH values in PNV and thick-choroid PCV patients compared to other groups.

Intra-class coefficient by the two graders for PP, MP, and FP area were 0.993, 0.940, and 0.943, respectively. The CVH values on UWICGA images of the total gradable area were 28.58 ± 4.97, 33.79 ± 8.15, 33.61 ± 11.50, 42.19 ± 13.25, and 43.59 ± 7.86 in controls and nAMD, thin-choroid PCV, thick-choroid PCV, and PNV patients, respectively (*p* < 0.001). CVH values in PNV and thick-choroid PCV patients were higher than those in other groups (all *p* ≤ 0.006). No significant difference was found among the control, nAMD, and thin-choroid PCV groups (all *p* ≥ 0.073). The ratios of each area to the total area were 0.06 ± 0.01, 0.60 ± 0.05, and 0.34 ± 0.05 for the PP area, MP, and FP, respectively (*p* < 0.001). A similar trend in CVH of the PP area and MP was found. CVH values of the total area, PP area, MP, and FP are summarized in Table 2 and Figure 3.

### 3.3. Other Choroidal Parameters Measured on UWICGA and OCT Images by Group

The subjective CVH grades reported by retina specialists were 0.31 ± 0.52, 0.66 ± 0.72, 1.03 ± 0.68, 1.92 ± 0.73, and 2.24 ± 0.47 in controls and nAMD, thin-choroid PCV, thick-choroid PCV, and PNV patients, respectively (*p* < 0.001). The intraclass correlation coefficient (ICC) for CVH grading between the graders was 0.829 (range, 0.762–0.879). 

CVDs on UWICGA images of the total gradable area were 0.26 ± 0.03, 0.27 ± 0.04, 0.26 ± 0.04, 0.29 ± 0.05, and 0.30 ± 0.04 in controls and nAMD, thin-choroid PCV, thick-choroid PCV, and PNV patients, respectively (*p* = 0.001). CVDs in the PNV and thick-choroid PCV groups were higher compared to in the other groups (all *p* ≤ 0.031). CVDs in the PNV and thick-choroid PCV groups did not differ (*p* = 0.895). The SFCT values were 210.28 ± 70.25, 192.64 ± 70.67, 249.37 ± 46.89, 330.39 ± 26.4, and 369.88 ± 69.62 in controls and nAMD, thin-choroid PCV, thick-choroid PCV, and PNV patients, respectively (*p* < 0.001). SFCT values were higher in the order of PNV, thick-choroid PCV, thick-choroid PCV, and nAMD patients, respectively (all *p* ≤ 0.031). Subjective CVH grades; CVDs of the total area, PP area, MP, and FP; and SFCT values are summarized in Table 3 and Supplementary Figures S1 and S2.

### 3.4. Correlation among Parameters

**Figure 4:**
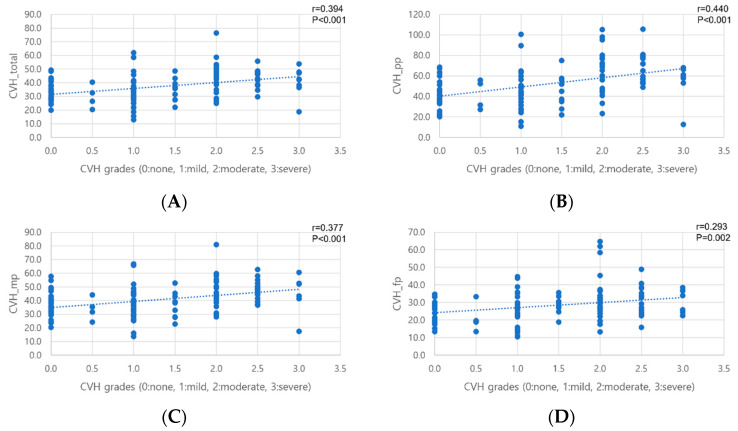
Correlation between manual choroidal vascular hyperpermeability (CVH) grades and measured CVH values. Measured CVH values positively correlated with manual CVH grades in the total gradable area (**A**), posterior pole area (**B**), mid-periphery (**C**), and far-periphery (**D**) areas.

Measured CVH positively correlated with subjective CVH grading of the total gradable area, PP area, MP, and FP (r = 0.394, r = 0.440, r = 0.377, and r = 0.293, respectively; all *p* ≤ 0.002, Figure 4). There were also positive correlations among CVH values of the total area, PP area, MP, and FP (all *p* < 0.001). CVH values of the total area and MP showed positive correlations with CVDs of the total area, PP area, and MP (all *p* ≤ 0.008). CVH values of all areas were positively correlated with SFCT (all *p* ≤ 0.003). Correlations between parameters are summarized in Table 4.

## 4. Discussion

Although CVH is an important finding in macular diseases, especially in some patients who present with macular CNVs, including nAMD, PCV, and pachychoroid spectrum diseases, the lack of objective measurement has limited its clinical application. In this study, we developed an objective quantifying method for CVH, compared the method with subjective grading by retina specialists, and compared the values among disease subtypes. 

The objective CVH measurement correlated well with the subjective CVH grades reported by the experts. The ICC between the graders was 0.829, and the correlation coefficient with the subjective CVH differed between graders, which can be referred to as subjectivity of the grading. The objective measurement of CVH can reduce these discrepancies between measurements by standardizing and automatizing the measurement procedure.

The comparison of CVH values between macular CNV subtypes revealed differences between diseases with and without pachychoroid phenotypes (i.e., nAMD and thin-choroid PCV vs. thick-choroid PCV and PNV). Along with anatomical differences in choroidal vasculature proven by OCT image analyses, functional differences between the subtypes were evidenced by this quantitative comparison [9,10]. The result is similar to that of Sasahara et al., who previously reported that the occurrence of CVH is more frequent in eyes with PCV than in those with AMD [3]. CVH is considered to result from leakage by weakened tight junctions of the choriocapillaris. Therefore, higher CVH values in thick-choroid PCV or PNV cases imply that more choriocapillaris leakage is involved in the pathophysiology of these eyes. The difference in treatment response according to CVH presence reported in other studies also supports this hypothesis [11,12].

CVH correlates with anatomical parameters, CVD, and SFCT. This result is in accordance with those of numerous previous studies. An association between CVH and choroidal venous congestion has been suggested since the 1990s [13]. Jirarattanasopa et al. reported higher SFCT values in eyes with CVH compared to eyes without CVH [14]. The association between choroidal vascularity on OCT B-scan images, SFCT, and CVH was reported by Lui et al. [15]. Additionally, Ryu et al. described a correlation between presence of CVH on UWICGA and CVD on OCT, and Jeong et al. found correlation between CVH on UWICGA and vortex vein engorgement in PCV eyes [16,17]. This study demonstrated global and quantitative correlations among these factors. The association between SFCT, CVD, and CVH values suggest that functional changes in the choriocapillaris relate to the anatomical changes in choroidal large vessels. 

In our study, mid-periphery was the area that best correlated with the total area in terms of CVD and CVH. This is because the MP is not only the largest area but also the area least affected by artifacts from macular exudation, hemorrhage, or peripheral distortion. In addition, widespread choroidal thickening and abnormal mid-peripheral fundus autofluorescence associated with CVH were previously reported by Nomura et al. [18]. In contrast, the correlation between CVH and CVD in the FP was not statistically significant. Image distortion and artifacts are more common in the peripheral areas of an image. Therefore, the interpretation of FP data requires caution. 

The study has some limitations including relatively small sample size for each subgroup. First, measurements were performed using one specific machine (Optos California P200DTx icg), and the CVH value can vary between ICGA systems. Nonetheless, application of the standardized method and subjective measurement in other machines is possible. Second, the image-processing methods, such as binarization, shadow compensation and contrast adjustment, used in the current study were selected as the best options after trialing numerous different methods. However, CVH and CVD values can differ if different image-processing methods are used, and there might be better untested methods. Third, since the focus of this study was to quantify CVH in macular diseases with CNV, thereby the object missed CSC, a disease which also shows prominent CVH on ICGA. A further study is warranted to validate this method in CSC eyes. In addition, this study used a cutoff value of arbitrary 300 μm to stratify thin- and thick-choroid PCV cases. This cutoff is not an absolute number for subtyping the disease, which should be considered when interpreting the results of this study. Despite these limitations, this study has value as the first quantitative analysis of CVH on UWICGA images.

In conclusion, the CVH parameter was measured objectively using quantitative UWICGA analysis and correlated with subjective CVH measurements reported by experts. CVH values differed among macular CNV subtypes and correlated with CVD and SFCT.

## Figures and Tables

**Table 1:** Demographics of the study subjects.

Variables	Controls	nAMD	Thin-Choroid PCV	Thick-Choroid PCV	PNV	*p* Value *
Case Numbers	18	25	19	18	33	
Age, years (mean ± SD)	69.22 ± 7.5	72.44 ± 12.95	70.63 ± 8.25	69.94 ± 6.13	64.12 ± 12.35	0.036 ^a^
BCVA, logMAR (mean ± SD)	0.36 ± 0.66	0.48 ± 0.6	0.44 ± 0.5	0.48 ± 0.34	0.22 ± 0.23	0.198 ^a^
Sex, male (n, %)	10 (56)	7 (28)	13 (68)	11 (61)	21 (64)	0.040 ^b^
Laterality, right eye (n, %)	11 (61)	13 (52)	14 (74)	8 (44)	19 (58)	0.448 ^b^

*Abbreviations:* nAMD, neovascular age-related macular degeneration; PCV, polypoidal choroidal vasculopathy; PNV, pachychoroid neovasculopathy; SD, standard deviation; BCVA, best-corrected visual acuity. * Comparison among groups. ^a^ By analysis of variance. ^b^ By chi-squared test.

**Table 2:** Choroidal vascular hyperpermeability (CVH) measurement by groups.

CVH Area	Controls	nAMD	Thin-Choroid PCV	Thick-Choroid PCV	PNV	*p* Value *
Total (pixel intensity, mean ± SD)	28.58 ± 4.97	33.79 ± 8.15	33.61 ± 11.50	42.19 ± 13.25	43.59 ± 7.86	<0.001
PP (pixel intensity, mean ± SD)	35.42 ± 10.62	44.56 ± 14.02	45.93 ± 19.74	61.5 ± 23.73	65.34 ± 15.33	<0.001
MP (pixel intensity, mean ± SD)	31.45 ± 5.67	38.36 ± 9.45	36.22 ± 12.38	46.53 ± 14.76	47.18 ± 8.31	<0.001
FP (pixel intensity, mean ± SD)	23.31 ± 5.22	25.7 ± 7.12	24.83 ± 9.19	30.23 ± 11.61	32.95 ± 10.18	0.001

*Abbreviations:* nAMD, neovascular age-related macular degeneration; PCV, polypoidal choroidal vasculopathy; PNV, pachychoroid neovasculopathy; SD, standard deviation; PP, posterior pole; MP, mid-periphery; FP, far-periphery. * Comparison among groups by analysis of variance.

**Table 3:** Choroidal vascular density (CVD) measurement by groups.

CVD Area	Controls	nAMD	Thin-Choroid PCV	Thick-Choroid PCV	PNV	*p* Value *
Total (pixel intensity, mean ± SD)	0.26 ± 0.03	0.27 ± 0.04	0.26 ± 0.04	0.29 ± 0.05	0.30 ± 0.04	0.001
PP (pixel intensity, mean ± SD)	0.31 ± 0.07	0.29 ± 0.09	0.30 ± 0.06	0.32 ± 0.08	0.38 ± 0.08	<0.001
MP (pixel intensity, mean ± SD)	0.26 ± 0.04	0.28 ± 0.05	0.26 ± 0.04	0.30 ± 0.05	0.30 ± 0.05	0.006
FP (pixel intensity, mean ± SD)	0.23 ± 0.04	0.25 ± 0.04	0.24 ± 0.04	0.27 ± 0.05	0.27 ± 0.05	0.026
Manual grades (score, mean ± SD)	0.31 ± 0.52	0.66 ± 0.72	1.03 ± 0.68	1.92 ± 0.73	2.24 ± 0.47	<0.001
SFCT (um, mean ± SD)	210.28 ± 70.25	192.64 ± 70.67	249.37 ± 46.89	330.39 ± 26.4	369.88 ± 69.62	<0.001

*Abbreviations:* nAMD, neovascular age-related macular degeneration; PCV, polypoidal choroidal vasculopathy; PNV, pachychoroid neovasculopathy; SD, standard deviation; PP, posterior pole; MP, mid-periphery; FP, far-periphery. * Comparison among groups by analysis of variance.

**Table 4:** Correlation between parameters.

CVH Area		CVH				CVD				SFCT	BCVA
		Total	PP	MP	FP	Total	PP	MP	FP		
Total	Correlation coefficient	1.000	0.887 **	0.971 **	0.806 **	0.305 **	0.247 **	0.250 **	0.310 **	0.330 **	−0.113
	*p*		<0.001	<0.001	<0.001	0.001	0.008	0.008	0.001	<0.001	0.233
PP	Correlation coefficient	0.887 **	1.000	0.904 **	0.533 **	0.376 **	0.336 **	0.295 **	0.399 **	0.387 **	−0.096
	*p*	<0.001		<0.001	<0.001	<0.001	<0.001	0.002	<0.001	<0.001	0.312
MP	Correlation coefficient	0.971 **	0.904 **	1.000	0.665 **	0.343 **	0.271 **	0.273 **	0.371 **	0.302 **	−0.100
	*p*	<0.001	<0.001		<0.001	<0.001	0.004	0.003	<0.001	0.001	0.290
FP	Correlation coefficient	0.806 **	0.533 **	0.665 **	1.000	0.106	0.078	0.107	0.075	0.226 *	−0.100
	*p*	<0.001	<0.001	<0.001		0.263	0.413	0.261	0.431	0.016	0.291

*Abbreviations:* CVH, choroidal vascular hyperpermeability; CVD, choroidal vessel density; SFCT, subfoveal choroidal thickness; BCVA, best-corrected visual acuity; PP, posterior pole; MP, mid-periphery; FP, far-periphery. ** Significant correlation of *p* < 0.005. * Significant correlation of *p* < 0.05.

## Data Availability

The data presented in this study are available on request from the corresponding author. The data are not publicly available due to hospitals data sharing policy.

## Supplementary Materials

The following supporting information can be downloaded at: https://www.mdpi.com/article/10.3390/diagnostics14070754/s1, Figure S1: Choroidal vascular density (CVD) measured on ultra-widefield indocyanine green angiography (UWICGA) images by group. Figure S2: Subfoveal choroidal thickness (SFCT) and subjective choroidal vascular hyperpermeability (CVH) grades by group.
