# Supplementary material for: Quantification of Choroidal Vascular Hyperpermeability on Ultra-Widefield Indocyanine Green Angiography in Macular Neovascularization
Source: Diagnostics (Basel). 2024 Apr 2;14(7):754. doi: 10.3390/diagnostics14070754 (PMC11011650; doi:10.3390/diagnostics14070754)
Supplement: Supplementary file 1 [file diagnostics-14-00754-s001.zip › diagnostics-2919399-supplementary.pdf]

Supplementary Materials

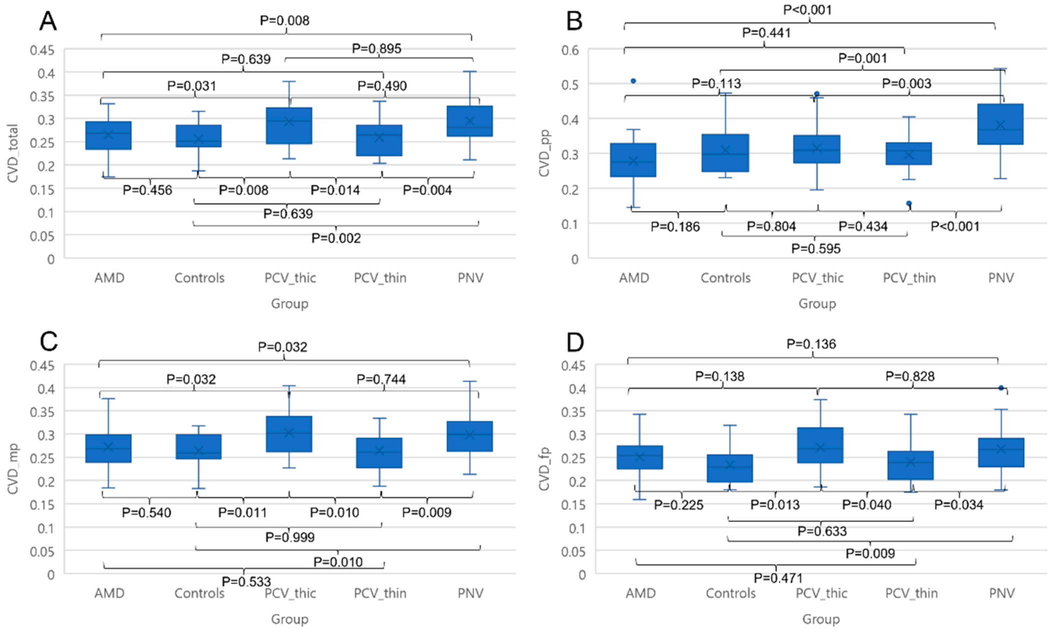

Figure S1. Choroidal vascular density (CVD) measured on ultra-widefield indocyanine green angiography (UWICGA) images by group. CVD on UWICGA images of the total gradable area (A), posterior pole (PP) area (B), mid-periphery (MP) (C), and far-periphery (FP) (D). UWICGA images of the total gradable area, PP, and MP revealed higher CVH values in PNV and thick-choroid PCV patients compared to other groups.

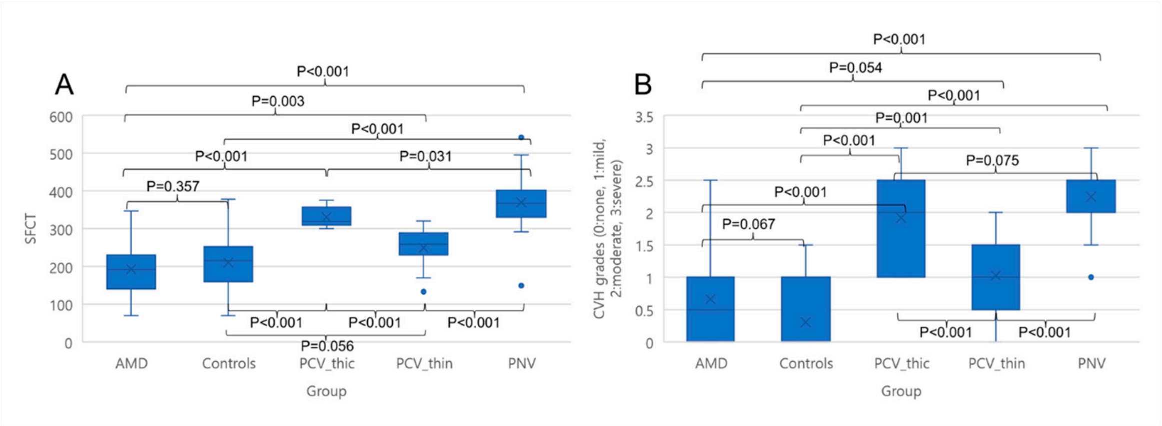

Figure S2. Subfoveal choroidal thickness (SFCT) and subjective choroidal vascular hyperpermeability (CVH) grades by group. (A) SFCT was highest in PNV patients followed by thick-choroid PCV, thin-choroid PCV, and nAMD patients. (B) Subjective CVH grades scored by 2 retina specialists were higher in thick-choroid PCV and PNV patients compared to the other groups.
